# Three‐dimensional organization of the cytoskeleton: A cryo‐electron tomography perspective

**DOI:** 10.1002/pro.3858

**Published:** 2020-05-28

**Authors:** Saikat Chakraborty, Marion Jasnin, Wolfgang Baumeister

**Affiliations:** ^1^ Department of Molecular Structural Biology Max Planck Institute of Biochemistry Martinsried Germany

**Keywords:** actin filaments, cryo‐electron tomography, in situ architecture, intermediate filaments, microtubules

## Abstract

Traditionally, structures of cytoskeletal components have been studied ex situ, that is, with biochemically purified materials. There are compelling reasons to develop approaches to study them in situ in their native functional context. In recent years, cryo‐electron tomography emerged as a powerful method for visualizing the molecular organization of unperturbed cellular landscapes with the potential to attain near‐atomic resolution. Here, we review recent works on the cytoskeleton using cryo‐electron tomography, demonstrating the power of in situ studies. We also highlight the potential of this method in addressing important questions pertinent to the field of cytoskeletal biomechanics.

**Abbreviations**
3D: three‐dimensional
ATP: adenosine triphosphate
CEMOVIS: cryo‐electron microscopy of vitreous sections
cryo‐EM: cryo‐electron microscopy
cryo‐ET: cryo‐electron tomography
EM: electron microscopy
ET: electron tomography
FH2: formin homology 2
FIB: focused ion beam
IFs: intermediate filaments
GDP: guanosine diphosphate
GTP: guanosine triphosphate
MAPs: microtubule‐associated proteins
MTs: microtubules
PFs: protofilaments
TAT: tubulin acetyltransferase
TEM: transmission electron microscopy
VASP: vasodilator‐stimulated phosphoprotein
VPP: Volta phase plate
WASP: Wiskott–Aldrich syndrome protein
WAVE: WASP‐family verprolin‐homologous protein

## 1 INTRODUCTION

In their seminal work on cellular mechanics, Francis Crick and Arthur Hughes proposed a model for the cytoskeleton: “If we were compelled to suggest a model (of cell mechanics) we would propose Mother's Work Basket—a jumble of beads and buttons of all shapes and sizes, with pins and threads for good measure, all jostling about and held together by colloidal forces.”^1^ In fact, around the middle of the 20th century, the idea gained momentum that the cell is not just passive material but a hierarchically ordered system of interacting molecular species (beads and buttons) and supramolecular entities (threads). Life depends on their interaction networks. Owing to advances in biochemistry and biophysics, we have now identified many of those beads and buttons, pins, and threads. These are the numerous cytoskeletal proteins, motor proteins as well as their regulatory molecules.

The cytoskeleton has three major constituents: actin filaments, microtubules (MTs), and intermediate filaments (IFs), which are organized into higher‐order assemblies. Each filament is structurally and functionally unique, interacts with its own set of proteins involved in filament crosslinking, bundling, capping, or severing, and performs specialized tasks within the cell. The regulation of the on–off kinetics of these interactors, the polymerization–depolymerization dynamics, polarity and chirality of the filaments, and the action of molecular motors is what makes the cytoskeleton a multifunctional supramolecular assembly.^2^ The traditional reductionist approach—studying one molecule at a time in isolation^3^—cannot lead to a comprehensive understanding of how cells use the cytoskeleton to perform exquisitely complex mechanical tasks such as division, locomotion, or shape remodeling.^4^ For example, weak or transient interactions between filaments or their binding partners are lost when the effects of macromolecular crowding are reduced.^5^ This might lead to nonphysiological conformational or oligomeric states, and their spatiotemporal modulation remains elusive when taken out of context.

**FIGURE 1:**
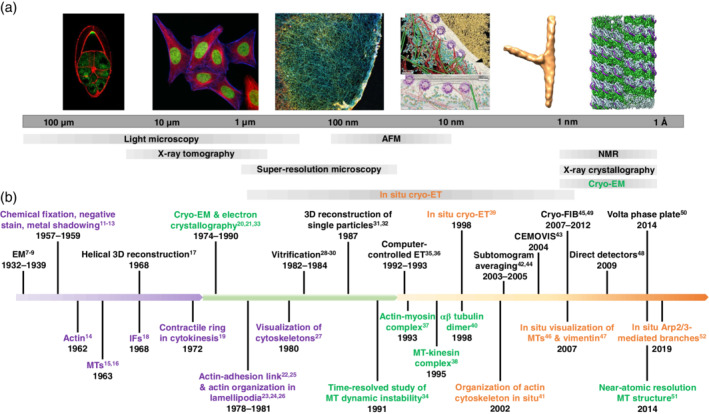
Evolution of the electron microscopy (EM) field with special emphasis on milestones in cytoskeleton research. (a) Biological length‐scales accessible through different imaging techniques. In situ cryo‐electron tomography (cryo‐ET; orange) spans length‐scales from several microns to the subnanometer range making it suitable for the exploration of cellular architecture and biomolecules. From left to right: A *Drosophila* oocyte stained for actin (red; courtesy of Dr. Daniel St. Johnston, University of Cambridge, UK), HeLa cells labeled for actin (blue), and microtubules (MTs; red), a Cos7 cell stained for actin (courtesy of Prof. Ralf Jungmann, LMU and MPIB, Germany), the nuclear periphery of a HeLa cell (reproduced with permission from reference [10], copyright (2016) AAAS), the in situ subtomogram average of Arp2/3 complex‐mediated branch junctions from *Dictyostelium discoideum* (EMD 4790) and the cryo‐EM reconstruction of the MT‐tau complex (EMD 7523). (b) The EM field is divided into three periods, starting from the invention of EM and the development of conventional sample preparation techniques (purple) to the emergence of single‐particle cryo‐EM (green) followed by in situ cryo‐ET (orange). The development of the EM field goes hand‐in‐hand with milestone discoveries of cytoskeletal elements and architectures highlighted with the same color (see references [7, 8, 9] and [11, 12, 13, 14, 15, 16, 17, 18, 19, 20, 21, 22, 23, 24, 25, 26, 27, 28, 29, 30, 31, 32, 33, 34, 35, 36, 37, 38, 39, 40, 41, 42, 43, 44, 45, 46, 47, 48, 49, 50, 51, 52])

The history of cytoskeletal research revolves around the pursuit of tools and techniques that promise an ever closer view of these elaborate structures. Studies in the 17th century using early microscopes revealed a network of fibrils in muscles^6^ and other tissue types. Since then, researchers strived to understand the three‐dimensional (3D) organization of cytoskeletal elements that forms the underlying robust, yet dynamic, scaffold of all cells. Along with it, the importance of establishing and maintaining order over different length‐ and time‐scales became apparent. With the emergence of electron microscopy (EM),^7^, ^8^, ^9^ unprecedented views of the cytoskeleton were provided (Figure 1). As EM techniques improved, they revealed an increasingly complicated cytoskeletal network in cells, composed of intermingled actin filaments, IFs and MTs, and numerous crosslinkers and associated proteins.^18^, ^19^, ^22^, ^23^, ^24^, ^25^, ^26^, ^27^ The major cytoskeletal elements, actin filaments,^14^ and MTs,^15^, ^16^ were first discovered in nonmuscle cells after the introduction of chemical fixation and heavy metal staining^13^ (Figure 1). However, this came with a price. Structural studies in this field have relied principally on two approaches: immunohistochemical techniques^53^, ^54^ and transmission electron microscopy (TEM) of plastic embedded or deep‐etched^11^ and metal‐shadowed specimens.^12^ First, some of the fixatives used in EM, although invaluable for the visualization of the cytoskeleton, are harsh compounds leading to structural alterations. Second, proteins associated with the cytoskeleton cannot be identified easily by these techniques. Although immuno‐EM provides a partial solution to the latter problem, the methodology is challenging and idiosyncratic. Fluorescence microscopy, when combined with cytoskeleton‐binding probes, offers a number of ways to highlight the cytoskeleton.^55^ However, the cytoskeleton is tightly packed, and it is not easy to discern individual structures owing to the diffraction limit of optical microscopy (approximately 300 nm). This issue has been resolved with the emergence of optical super‐resolution imaging techniques,^56^ although these techniques are still limited in resolution (~20–50 nm).^57^

Therefore, there was a need for a method that provides faithful representations of functional modules and their interplay in a cellular context. This can be achieved through structural studies performed in situ, that is, in unperturbed environments. Cryo‐electron tomography (cryo‐ET) fulfills these criteria: it provides molecular resolution information of cells and organelles unadulterated by specimen preparation.^58^, ^59^ Rapid freezing ensures the best structural preservation that is physically possible to achieve.^28^, ^29^, ^30^ Although the idea to use ET for native samples was there for decades,^60^ the realization of the vision followed only much later^39^, ^61^ (Figure 1). Technological advances such as computer‐controlled transmission electron microscopes made it possible to develop automated data acquisition procedures minimizing exposure to the electron beam.^35^, ^36^ The advent of focused ion beam (FIB) milling adapted to cryogenic conditions^45^, ^49^ permitted the reproducible preparation of thin vitrified cellular samples without the notorious artefacts of cryo‐sectioning such as sample compression.^62^ With the development of direct electron detection^48^ and advances in image processing,^17^, ^20^, ^21^, ^31^, ^32^, ^33^, ^42^, ^44^ we are now entering the realm of subnanometer resolution for structural studies of the cellular interior.^58^ Cryo‐ET technologies have already started to provide new insights into the 3D architecture of the cytoskeleton in situ (Figure 1). In this review, we discuss recent progress toward a structural understanding of the cytoskeleton; in particular, we show how the application of in situ approaches has led to new insights into the organization and function of cytoskeletal filaments that had remained elusive so far.

## 2 THE ARCHITECTURE OF THE ACTIN CYTOSKELETON

The actin cytoskeleton is essential for motile cells to modulate their shape and move within complex environments. It adopts a variety of architectures that contribute to protrusion, adhesion, contraction, and retraction of the cell.^63^ At the leading edge, branched and crosslinked networks form a lamellipodium that, by pushing the plasma membrane forward, promotes cell movement.^64^ Thin actin‐rich, finger‐like membrane protrusions called filopodia assemble from peripheral regions of the cell in response to chemical stimuli, providing initial cell‐substrate contact sites.^65^, ^66^ At the basal cell membrane, self‐organized actin waves propagate,^67^ and, in invasive cells, large filopodia‐like protrusions called invadopodia can penetrate through the extracellular matrix.^68^ Podosomes extend a core of branched and crosslinked actin filaments into the cytoplasm for mechanosensing.^69^ Cell contractility arises from the association of actin with myosin II^37^ as exemplified in stress fibers, thick antiparallel bundles anchored at focal adhesion sites where they sense, generate, and transmit tension to the extracellular matrix.^70^ The actomyosin cortex lying beneath the plasma membrane contributes to changes and maintenance of cell shape.^71^ When membranes occasionally detach from the cortex and inflate, spherical protrusions, called blebs, are transiently generated; upon the reassembly of an actin cortex the blebs can be retracted.^72^

The assembly of the diverse cellular actin architectures is finely tuned by a large array of actin‐associated proteins. Actin nucleation and elongation factors comprise the Arp2/3 complex, formins, and Ena/vasodilator‐stimulated phosphoprotein (VASP), which generate branched or linear filaments, respectively.^63^ Several bundling and crosslinking proteins, including fascin, fimbrins, alpha‐actinins, and filamins, can connect filaments over a wide range of distances, contributing to the macroscale organization of the networks.^63^ In vitro studies are key to decipher the architectural properties of actin arrays arising from a defined pool of accessory proteins. Visualizing actin network architectures within the complex cellular environment is essential to understand physiological higher‐order actin assemblies in relation to actin function. Since the first in situ observation of the actin cytoskeleton by Medalia et al. almost two decades ago,^41^ the cryo‐ET field has evolved considerably through a series of technological developments that include direct electron detectors,^48^ cryo‐FIB milling,^45^, ^49^, ^73^ and Volta phase plates (VPPs).^50^ In the following sections, we summarize what we have learnt so far about the nanoscale organization of actin bundles and branched networks inside cells using cryo‐ET or ET. Additionally, we discuss the ultrastructure of two composite actin networks, *Listeria* comet tails, and self‐organized actin waves.

### 2.1 *Nanoscale organization of cellular actin bundles*

Before the emergence of cryo‐FIB milling sample preparation, cryo‐ET was limited to thin peripheral parts of the cell. Cryo‐ET could be employed to show the organization of filopodia in *Dictyostelium* cells.^74^ The resolution (4–6 nm) at the time was sufficient to resolve individual actin filaments (diameter of 7–10 nm) within the tomograms. Using a correlative approach, Patla et al. examined chemically fixed actin bundles associated with integrin‐mediated focal adhesion sites at the periphery of REF52 cells.^75^ They observed flat pseudopodial protrusions filled with unidirectional actin bundles and adhesion complexes.

**FIGURE 2:**
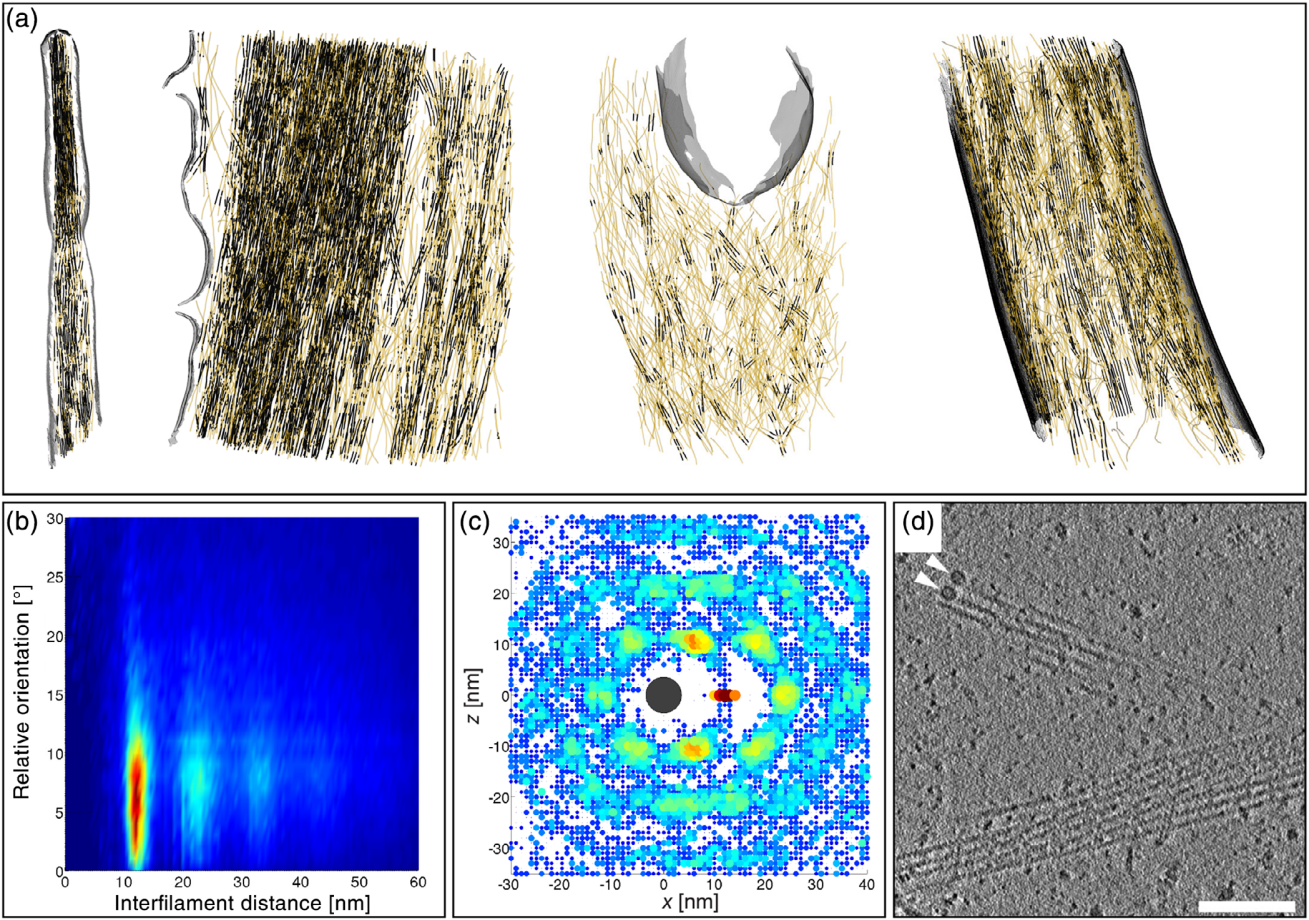
Nanoscale architecture of cellular actin bundles explored by cryo‐electron tomography. (a) Three‐dimensional (3D) architecture of peripheral actin networks in Ptk2 cells. From left to right: actin filaments in a filopodium, a stress fiber, a cytoplasmic *Listeria* comet tail, and a *Listeria* protrusion. Actin filaments are shown in yellow with bundled portions displayed in black. The plasma membrane and the cell wall of *Listeria* are shown in grey. (b) 2D histogram showing distances between filopodial actin filaments as a function of their relative orientation, and revealing a population of parallel filaments with center‐to‐center distances of ~12 nm. Second‐ and third‐order peaks are visible indicating long‐range order. Reproduced from reference [77]. (c) Packing analysis for actin filaments belonging to a filopodium revealing a hexagonal arrangement of neighboring filaments. Reproduced from reference [77]. (d) Slice through a tomogram of a neuronal process acquired with the Volta phase plate. Two annular structures (white arrowheads) resembling formin homology 2 domains are visible at the tip of actin filaments. The periodicity of the helical repeat of actin filaments is evident in the lower bundle. Scale bar: 100 nm. Reproduced with permission from reference [78], copyright (2015) Elsevier

This initial work lead to the development of an automated segmentation method allowing for robust description of filament networks.^76^ The method is based on template matching of a generic filament, providing cross‐correlation maps for the extraction of the filament centerlines, which can either be used for visualization purposes or for a quantitative description of the networks. Taking advantage of this automated segmentation procedure, methodologies were developed to quantitatively analyze the nanoscale architecture of filopodia, stress fibers, and *Listeria* comet tails imaged at the periphery of intact Ptk2 cells using cryo‐ET.^77^ The study revealed the existence of nanoscopic bundles within these networks, with interfilament distances of 12–13 nm, suggesting that nanoscopic bundles are a generic feature of actin assemblies involved in motility (Figure 2a,b). In filopodia and *Listeria* protrusions, actin bundles are hexagonally packed (Figure 2c), whereas in stress fibers and cytoplasmic comets tails, they rather form sheets. The data further indicate that bundling proteins, fascin, and fimbrin are commensurate with a narrow range of interfilament distances and allow for hexagonal packing. In *Dictyostelium* cells, short bundles of actin filaments persist during membrane pearling induced by actin depolymerization using latrunculin A.^79^

Combining direct detection with VPP imaging, Fukuda et al. pushed the frontiers of quality for tomographic images of actin filaments in primary neurons.^78^ The resolution (better than 3 nm) permitted to resolve the periodicity of actin filaments. They visualized two annular structures (~11 nm in size) at the end of actin filaments forming a hexagonal bundle (Figure 2d). These densities are likely associated with the formin homology 2 (FH2) domain responsible for the binding of formin to the barbed end of actin filaments.^80^, ^81^, ^82^ Future in situ cryo‐ET studies may help uncover the structure of the FH2 domain bound to an actin filament, and to explore how formins contribute to the generation of diverse cellular actin organizations.

Recently, photo‐micropatterning of EM grids enabled controlled positioning of cells with predictable actin organization.^83^, ^84^ By applying cryo‐FIB milling to a RP1E cell grown on a crossbow micropattern, actin transverse arcs and internal stress fibers could be targeted by cryo‐ET.^83^ This approach will be instrumental in future studies to unravel the relationship between cytoskeleton organization and biomechanics at the nanoscale.

### 2.2 *Classical branched organization mediated by Arp2/3 complex*

To date, most of the structural knowledge on cellular actin organization derives from studies on the lamellipodia of keratocytes and fibroblasts. First, conventional EM work on negatively stained fibroblasts suggested that the lamellipodium is made of long diagonally oriented actin filaments.^23^, ^24^, ^26^ Two decades later, platinum‐replica EM work showed that this flat (0.1–0.2 μm) cellular protrusion is made of branched filament arrays,^85^ contributing to the emergence of the dendritic nucleation model for the mechanism of actin assembly by Arp2/3 complex at the leading edge of the cell.^86^ The model proposes that upon recruitment and activation at the protruding cell membrane, the Arp2/3 complex initiates actin polymerization by branching off new filaments from the side of mother filaments at an angle of 70°.^87^, ^88^ Both mother and daughter filaments grow with their barbed ends facing the cell membrane with an incidence angle of 55° until they get capped.^86^, ^89^ Filament growth toward the membrane results in network expansion whereas disassembly deeper in the cell provides a pool of subunits for the growth of dendritic arrays.^86^ Years later, Small and coworkers argued that the visualization of branched networks by platinum‐replica EM is an artefact of critical‐point drying.^90^ They challenged the dendritic nucleation model for lamellipodia propulsion using ET data of vitreous and negatively stained lamellipodial networks, in which they found that filaments were mostly unbranched.^90^ However, using their primary ET data, Yang and Svitkina detected a significant number of branch junctions within one tomogram,^91^ later confirmed by Small et al.^92^

Recently, Mueller et al. used ET to quantify how the lamellipodium of fish keratocytes responds to varying forces associated with changes in membrane tension.^93^ They showed that the incident angle of the branched filaments with respect to the membrane varies with the load, from the typical 55° angle to 90° in case of reduced membrane tension. In contrast, an increased load on the lamellipodium produces a dense dendritic network with a broadened range of angles relative to the membrane. The load adaptation of the lamellipodium is a direct consequence of the nucleation geometry of branched networks at the membrane.^93^ A recent study showed that force generation in lamellipodia depends additionally on formin‐like protein (FMNL) formin activity.^94^ Future in situ cryo‐ET studies could help reveal the fine 3D architectural changes associated with the loss of specific formins and how this may result in changes in force production. More generally, they would provide valuable insights into the native 3D architecture of the lamellipodium, its transition to a lamella^95^ and how these two types of actin networks overlap during the protrusion of the cell.^96^

**FIGURE 3:**
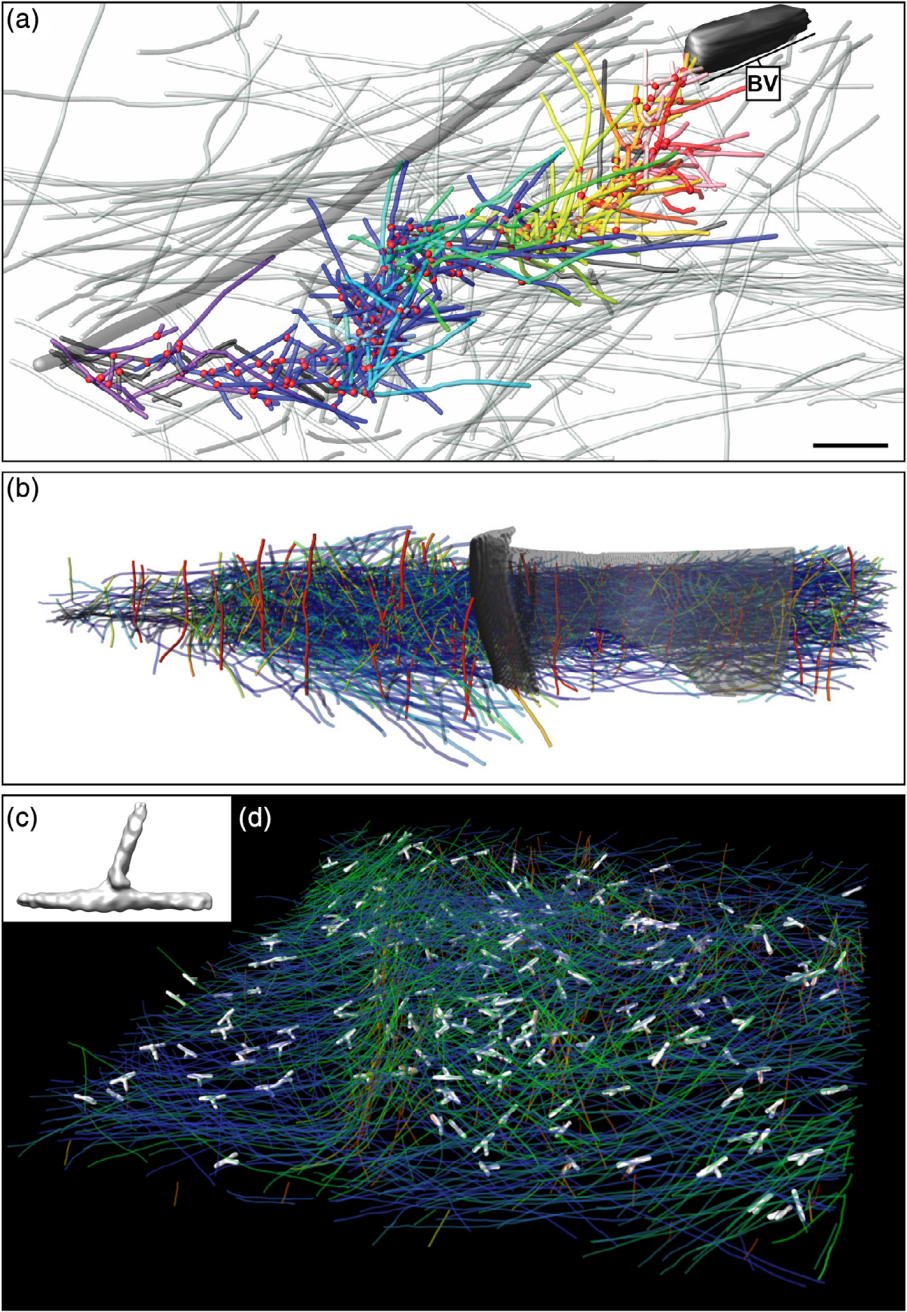
Branched actin structures revealed using cryo‐electron tomography. (a) Three‐dimensional (3D) architecture of a baculovirus (“BV”) actin tail assembled in a B16 melanoma cell. Branch points (red dots), actin filaments of the host cytoskeleton (translucent lines) and the comet tail (colored lines), and one microtubule (grey tube) are represented. Reproduced from reference [97]. (b) 3D organization of a *Listeria* comet tail assembled in *Xenopus* egg extracts. Actin filaments are represented in a blue‐to‐red color map as a function of their elevation angle relative to the support film. The cell wall of *Listeria* is shown in grey. Reproduced with permission from reference [98], copyright (2016) Elsevier. (c) Density map of branch junctions mediated by the Arp2/3 complex from *Dictyostelium* cells (EMD 4790) in solid‐surface representation. (d) Branch junctions (white) in an actin wave traveling at the basement membrane of *Dictyostelium* cells. Actin filaments are rendered in the same color as in (b)

Apart from the lamellipodium, ET permitted to reveal the architecture of baculovirus actin comet tails assembled by Arp2/3 complex.^97^ Mueller et al. showed that the small virus (~50 nm in diameter) assembles a fishbone‐like array of actin filaments generated by the formation of branch junctions (Figure 3a). They proposed a model for baculovirus propulsion based on branch tethering at the virus surface.

In addition to providing architectural insights into branched networks, ET was used to obtain the structure of reconstituted and negatively stained branch junctions of *Acanthamoeba castellanii* with a resolution of 2.6 nm showing a uniform branching angle of 78°.^99^ A similar structure was obtained from chemically fixed and negatively stained lamellipodia of NIH3T3 cells.^100^ Using cryo‐FIB milling, cryo‐CLEM and cryo‐ET, Jasnin and colleagues targeted actin waves traveling at the basal membrane of *Dictyostelium* cells.^52^ Template matching and subtomogram averaging^42^, ^44^ served to identify branch junctions in different orientations. The in situ structure of branch junctions obtained at a resolution of 3.1 nm (EMD 4790, Figure 3c) showed a conserved branch angle of 70° in agreement with previous in vitro measurements using electron and light microscopy^86^, ^101^ and ET of lamellipodia.^90^, ^91^ The angle differs from the uniform 78° angle obtained for the reconstituted amoeba samples,^99^ which may derive from the limited range of orientations contributing to the reconstruction. In higher eukaryotes, Arp2/3 complex is a family of complexes with a pleiotropic impact on actin filament dynamics.^102^ This raises the possibility that there exist Arp2/3 complexes inducing different branching conformations, a question that could be addressed through cryo‐ET. The work on actin waves paves the way for further in situ structural investigations of branched networks, and holds promise for obtaining a native high‐resolution structure of Arp2/3 complex‐mediated branch junctions.

### 2.3 *Listeria* comet tails and actin waves: Two examples of collaborative actin assembly

#### 2.3.1 Cytoplasmic tails and protrusions assembled by *Listeria monocytogenes*

*Listeria* movement and propagation in host cells is facilitated by the ability of the bacteria to hijack the Arp2/3 complex activity. *Listeria* expresses at its surface the transmembrane protein ActA, which recruits Arp2/3 complex, VASP, and G‐actin, and mimics the Wiskott‐Aldrich Syndrome protein (WASP)/WASP‐family verprolin‐homologous protein (WAVE) family of nucleation promoting factors.^103^ Cryo‐ET studies demonstrated that the ultrastructure of *Listeria* tails assembled inside cells^77^ and in *Xenopus* egg extracts^98^ (Figure 3b) is different from a classical dendritic organization as well as from the fishbone structure reported for the baculovirus tails^97^ (Figure 3a). Unlike lamellipodia and baculovirus tails, in *Listeria* comet tails, filament intersections showing a branch geometry are randomly oriented with respect to the polymerizing surface and the direction of movement.^98^ In addition, nanoscopic actin bundles were observed within the comets (Figure 2a,b), some of them hexagonally packed (Figure 2c), indicating that Arp2/3 complex alone does not determine the tail architecture.^98^ In a reconstituted motility system, increasing the ratio of VASP to Arp2/3 at the polymerizing surface induces filament alignment parallel to the direction of movement.^104^ The architecture of *Listeria* tails observed during intracellular motility and cell‐to‐cell spread may therefore reflect the combined action of Arp2/3 complex and VASP at the bacterial surface.^98^ Higher‐order organization of the aligned filaments into hexagonal bundles may arise from the diverse bundlers and cross‐linkers present in *Listeria* tails.^98^

Other viral and bacterial pathogens, including *Shigella flexneri*, *Rickettsia rickettsii*, and the *vaccinia* virus, have developed similar strategies to promote their dissemination into host cells.^105^ The structural diversity of actin tails assembled by intracellular pathogens illustrates the malleability of the actin machinery in generating a broad spectrum of architectures through fine differences in actin‐associated factors and nucleation geometry. Exploring pathogen subversion of host cell cytoskeletal machinery using in situ cryo‐ET can provide valuable insights into the molecular and structural basis of actin‐based motility.

#### 2.3.2 *Traveling actin waves*

Another phenomenon linked to composite actin networks is provided by the propagation of self‐organized actin waves at the cell cortex. Since their initial observation in *Dictyostelium* cells,^106^ actin waves were reported in a variety of motile cells and correlated with cell protrusion, polarization, migration, and adhesion.^107^, ^108^ Our understanding of these excitable dynamic actin patterns comes mostly from live‐imaging experiments and mathematical modeling. Recently, cryo‐ET has been used to explore the architecture of actin waves assembled at the basement membrane of *Dictyostelium* cells^52^ and how they respond to geometrical cues.^109^ These waves consist of oblique tent‐like arrays within a dense horizontal meshwork. Subtomogram averaging was used to identify Arp2/3 complex‐mediated branch junctions within the waves (Figure 3d). Spatial pattern analysis revealed that branch points cluster strongly in the waves. Most branches are nucleated from filaments oriented parallel to the membrane, and most daughter filaments grow either toward or parallel to the membrane. This differs from the classical dendritic organization observed in lamellipodia and associated with pure Arp2/3 complex nucleation, in which both mother and daughter filaments point toward the membrane at an angle of ~55°.^86^, ^89^ Live‐imaging data showed that VASP forms patches followed by actin polymerization at the wave front and associated with Arp2/3 complex clustering.^52^ This suggests that VASP generates at least part of the filaments from which Arp2/3 complex nucleates branches. In addition, formin B is localized to the actin waves^110^ and may complement VASP in supplying the Arp2/3 complex with mother filaments.

The wave architecture exemplifies a complex actin network organization derived from the combined action of actin nucleation and elongation factors. The variety of actin waves associated with diverse cell systems provides an opportunity to explore the versatile actin machinery at work and reveal the interplay of actin assembly factors, architecture, and function. Additionally, other composite cellular actin structures such as podosomes and phagocytic cups could help expand our understanding of actin assembly and force generation in situ.

## 3 NANOSCALE ARCHITECTURE OF THE MT CYTOSKELETON

The MT cytoskeleton constitutes the underlying skeletal framework that is crucial for many cellular functions including cell division,^111^, ^112^ motility,^113^ and cell architecture.^114^ MTs are polymerized filaments assembled of α‐ and β‐tubulin monomers in a head‐to‐tail arrangement which organize into a hollow cylinder with a diameter of 25 nm.^40^, ^51^, ^115^ Conventional EM with stained MTs has been instrumental in studying filaments formed by tubulins in cellular sections.^15^ Once cryo‐EM was developed as a near‐physiological sample preparation method, cryo‐EM images of reconstituted MTs yielded key insights into their nucleotide‐dependent dynamics and their interactions with their binding partners including molecular motors.^38^, ^116^

In order to relate MT architecture to its dynamics and function, it is necessary to elucidate MT structure along with its interacting proteins in their functional context. Early tomographic studies of MTs were performed on intact neuronal cells,^117^ fibroblasts,^118^ and vitreous sections of animal cells.^46^ They revealed several novel structural features: (a) MT lumen is not empty but often contains electron dense particles; (b) MT plus ends are mostly frayed structures and differ from reconstituted plus ends; and (c) cellular MTs are not exactly cylindrically symmetric. In the following paragraphs, we will report some of these findings, with a special emphasis on cryo‐ET studies.

### 3.1 *Architecture of the MT cytoskeleton*

The architecture of the MT cytoskeleton depends on the cell type and physiological state. An early ET study provided insights into the organization of interphase MTs in plastic‐embedded *Schizosaccharomyces pombe* cells.^119^ Höög et al. obtained a 3D reconstruction of a whole interphase cell revealing a bundled MT organization.^119^ MT polarity was determined based on their plus end appearances, with most cytoplasmic MTs open at one end and capped at the other. Several connections between MTs and between MTs and the nuclear envelope were identified, the latter suggesting a role in organelle positioning.

The most elaborate MT architecture is found in mitotic cells in the form of a spindle. Mitotic spindle organization is vital for chromosome segregation.^120^ Yet, the structure–function relationship of a spindle has remained largely unexplored. Ward et al. performed ET of fission yeast spindles, revealing that structural integrity comes from MT organization into a rigid hexagonally packed transverse array.^121^ Such arrangement maximizes the interactions between a MT and its neighbors, making the spindle stronger and preventing it from buckling under compressive forces. However, structural integrity of MTs was found to be compromised in the spindle of the smallest eukaryote, *Ostreococcus tauri*.^122^ Using cryo‐ET of high‐pressure frozen cells, Gan et al. found that spindle MTs in this organism are short and mostly incomplete (C‐shaped cross‐sections), comprising fewer than 13 protofilaments (PFs).^122^

**FIGURE 4:**
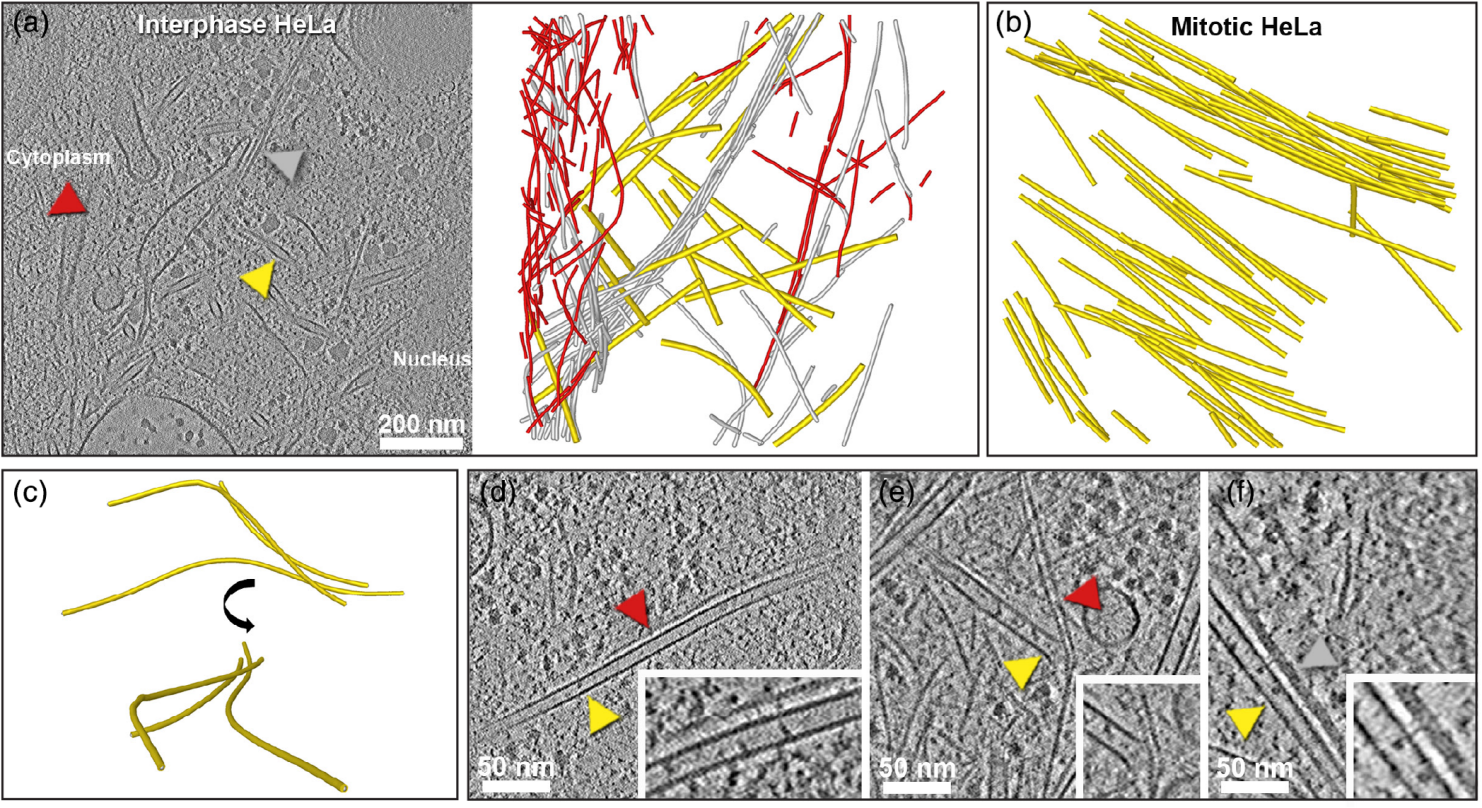
Architecture of the microtubule (MT) cytoskeleton revealed by cryo‐electron tomography. (a) Slice through a tomogram acquired in a focused ion beam‐milled interphase HeLa cell with the Volta phase plate. Arrowheads indicate a MT (yellow), an intermediate filament (IF; grey), and an actin filament (red). Cytoskeletal filaments were segmented and displayed using the same color code on the right panel. (b) Bundled organization of MTs in a mitotic HeLa cell. (c) Curvilinear trajectories of MTs in an interphase HeLa cell resembling short wavelength buckling and shown in two different orientations. (d) MT‐actin wall‐to‐wall interaction in an interphase HeLa cell. Inset: Close up showing putative crosslinkers connecting the filaments. (e) Actin‐MT plus end interaction in an interphase P19 cell. Inset: Zoomed image of the interaction zone. (f) MT‐IF wall‐to‐wall interaction in an interphase HeLa cell. Inset: Close up showing putative thin stalk‐like densities connecting the filaments. Panels (a) and (d‐f) reproduced from reference [123]

Recently, cryo‐FIB milling and cryo‐ET were harnessed to explore the nanoscale architecture of MTs inside different mammalian cells and at different cell‐cycle stages using the VPP.^123^ In interphase HeLa cells, MTs are spatially entangled in a composite cytoskeletal matrix of actin filaments and IFs (Figure 4a). Similar organization was found for astral MTs at the cortex of mitotic cells. Further away from the cortex, mitotic cells have a cytoskeletal organization dominated by the high abundance of parallel arrays of MTs forming a spindle (Figure 4b), with an interfilament distance of 65 nm. Smaller distances (~50 nm) were observed in MT bundles assembled in thin cellular parts of neuronal cells. In the presence of Taxol, the distance between parallel MTs in both mitotic and interphase HeLa cells is reduced.

### 3.2 *MT curvature in situ*

MT curvature plays a crucial role for biological processes which require the MT cytoskeleton to function as a scaffold. MTs need to be sufficiently stiff to create and maintain cell shape, especially for extended morphologies such as neuronal processes. MTs also need to remain fairly straight to function as long‐range tracks for efficient molecule and organelle transport by cargo‐carrying motor proteins. Consistent with this, isolated MTs have persistence length on the order of a 1–6 mm,^124^ whereas typical mammalian cells measure only tens of microns.

However, MTs were occasionally found in highly bent configurations inside cells.^125^, ^126^ In a recent cryo‐ET study, MT curvature was quantitatively analyzed inside interphase and mitotic cells.^123^ The apparent persistence length of MTs in interphase HeLa cells is ~2–3 orders of magnitude lower than their in vitro persistence length, consistent with previous reports based on fluorescence microscopy investigations.^127^, ^128^ In a subset of MTs residing at the nucleocytoplasmic boundary of HeLa cells, highly localized bends with sinusoidal wave‐like trajectories (named “short wavelength bending”) were found (Figure 4c). This contradicts the long‐arc‐like bending predicted by classical mechanics.^129^ Astral MTs entangled with actin filaments and IFs at the cortex of mitotic cells have similar curvatures as MTs in interphase cells. In contrast, spindle and neuronal MTs show long‐arc‐like bending. Apparently, the matrix surrounding the MTs can substantially modulate the curvature.^123^

### 3.3 *MT‐actin crosstalk*

Biophysical and biological properties of the actin and MT cytoskeletons have been studied extensively over the past decades. It is accepted that their functional dynamical properties are intimately intertwined. Actin‐MTs crosstalk is known to promote symmetry‐breaking polarization for cell shape changes, division, and motility.^130^ Early observations with conventional TEM revealed that MTs and actin filaments interact in the presence of MT‐associated proteins (MAPs) ex situ^131^ and in situ.^132^ Yet, how these two cytoskeletal systems work together during cellular processes remains to be elucidated.

In situ cryo‐ET of eukaryotic cells provided insights into MT and actin coordination inside cells.^123^ MTs and actin filaments were found to interact primarily through passive entanglements (Figure 4a). Such entanglements are known to create steric interactions between the two types of filaments inside the cytosol, and thereby influence cell shape and mechanics by restraining filament buckling and coordinated reinforcement of the cytoskeleton.^133^ Indeed, the ability of MTs to withstand strong compressive forces relies on the reinforcement provided by the surrounding elastic actin network that constrains MT bending.^126^ MTs were also found to interact with actin filaments via crosslinkers, either through wall‐to‐wall interactions with a spacing of about 11 nm (Figure 4d) or via their plus ends (Figure 4e).^123^ The latter interaction is very common at the actomyosin cortex, providing a physical barrier that prevents growing MTs from targeting the plasma membrane^130^ and can induce MT bending and catastrophe.^134^ It is also involved in actin‐mediated MT guidance and growth along actin bundles.^135^ The physical connections could be mediated by proteins with binding sites for both the actin filaments and MTs or MT‐end binding proteins such as CLIP‐170.^136^ Visualization of both types of interactions using in situ cryo‐ET supports the tensegrity model, in which tension propagation requires physical crosslinks between actin filaments and MTs.^137^

### 3.4 *Insights into the native MT structure*

**FIGURE 5:**
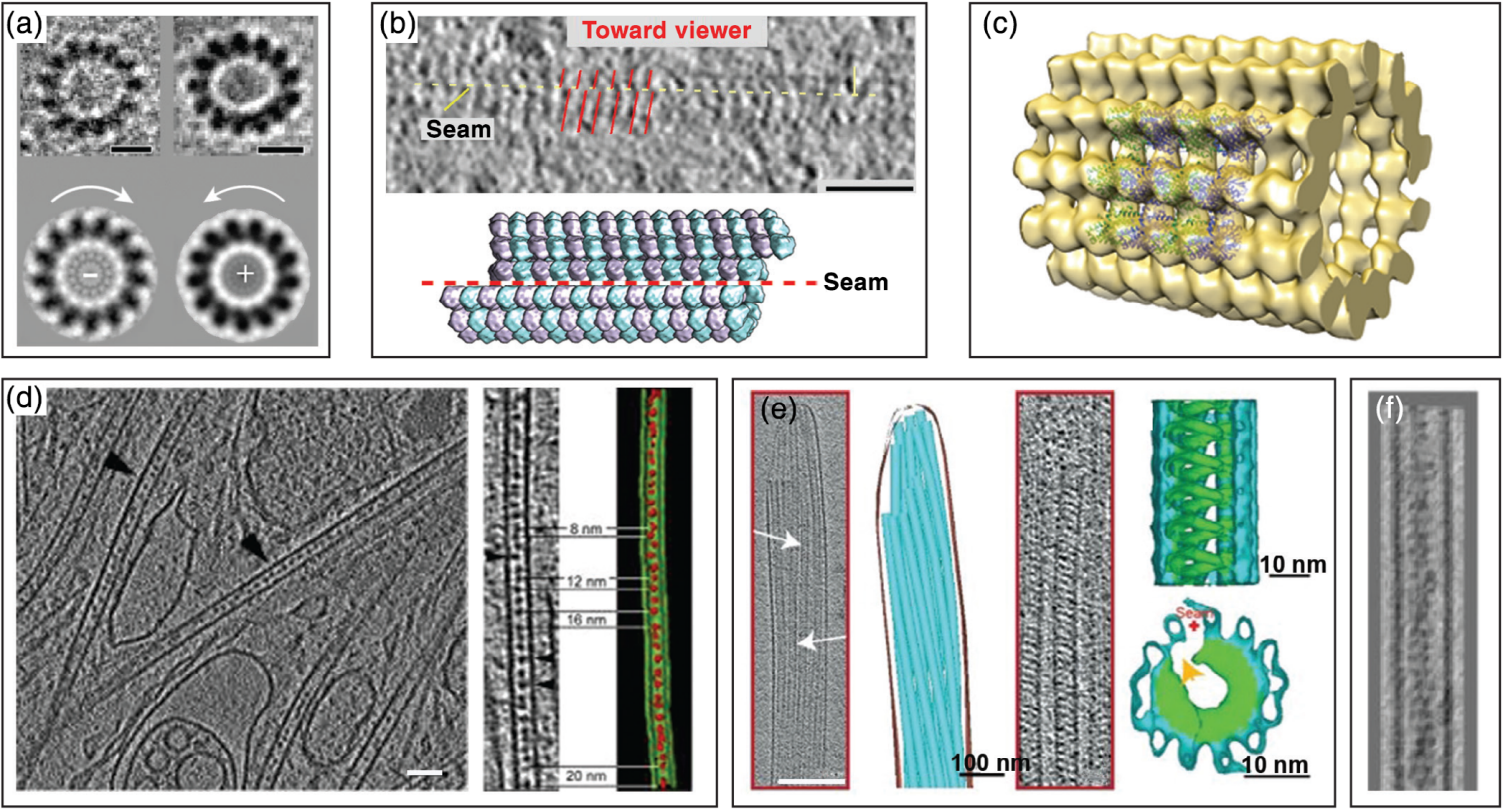
Native microtubule (MT) structure revealed by cryo‐electron tomography. (a) Cross‐sections through MTs in CHO cells (top) and “clockwise slew” (bottom left) or “anticlockwise slew” (bottom right) structure obtained by 13‐fold rotational averaging revealing MT polarity. Scale bars: 10 nm. Reproduced with permission from reference [46], copyright (2012) John Wiley and Sons. (b) Seam observed in cytoplasmic MTs decorated with Eg5 motor domains in an interphase 3T3 cell. Scale bar: 50 nm. Reproduced with permission from reference [141], copyright (2009) Elsevier. (c) MT structure of U87MG neuronal cells obtained by subtomogram averaging, with the fit of a MT segment (EMD 6O2R) in the density map. The structure confirmed the presence of 13 protofilaments in situ. (d) Heterogeneous lumenal densities observed in MTs of neuronal cells. Right: Close up in the MT lumen showing segmented material and interparticle distances. Scale bar: 100 nm. Reproduced with permission from reference [117], copyright (2006) Rockefeller University Press. (e) Singlet MTs observed at the tip of a sperm cell containing diagonal lumenal densities with 8 nm periodicity. Density map of the tail axoneme intra‐lumenal spirals complex obtained by subtomogram averaging (right). Reproduced from reference [142]. (f) Actin filament segments in the MT lumen of MT‐based cellular projections in HAP1 cells. Reproduced from reference [143]

In vitro‐assembled tubulins are known to yield polymorphic MT structures with PFs varying between 11 and 18.^138^, ^139^ Early conventional EM studies in 1970s using glutaraldehyde fixation and negative staining have shown that MTs in animal cells are mostly made of 13 PFs.^140^ Using cryo‐electron microscopy of vitreous sections (CEMOVIS)^43^ and cryo‐ET, Bouchet‐Marquis et al. explored MT ultrastructure in vitreous sections of high‐pressure frozen CHO cells.^46^ MT chirality was visible in the raw data and enhanced by 13‐fold rotational averaging, revealing a “clockwise slew” (structure seen from the minus end) or an “anticlockwise slew” (structure seen from the plus end; Figure 5a). The authors showed that tomography of vitreous sections can achieve a similar resolution as with plunge‐frozen samples, despite the compression artifacts associated with the cutting process. The same MT structure was later observed in thin cellular peripheries of primary fibroblasts and neurons using cryo‐ET.^118^ When McIntosh et al. visualized Eg5‐decorated interphase MTs in 3T3 cells using cryo‐ET, visual inspection of the tomograms and power spectra of the single‐projection images confirmed the presence of B‐lattices, in which the α‐tubulins of one PF lie next to α‐tubulins in the neighboring PFs^141^ (Figure 5b). As a result, 13‐PFs MTs with B‐lattices must include a “seam,” that is, a pair of PFs where α‐tubulin lies beside β‐tubulin, and therefore breach cylindrical symmetry (Figure 5b).

Cryo‐ET also offers an unbiased way to determine 3D structures using subtomogram averaging.^42^, ^44^ MT structure was elucidated in situ at 18–24 Å from a single tomogram^144^ (Figure 5c). The resulting MT structure is consistent with the current understanding of MT structure in vivo. Given the recent technical advancements, cryo‐ET will be the method of choice to generate in situ structures of MTs with their binding partners routinely, at resolutions facilitating a mechanistic understanding of MT‐related functions.

### 3.5 *Lumenal densities*

MAPs contribute to the stability of MTs and regulate their dynamics whereas MT‐based motors mediate the transport of cargoes along MT tracks. Although all MAPs and motors studied so far bind to the outside of the MT wall, small molecules such as Taxol can associate with the lumenal side of MTs.^145^ The MT lumen is physically separated from the cytoplasm, except from the lateral pores and two 200 nm^2^ entrances at the MT ends. The presence of electron‐dense material within the MT lumen was first observed in plastic‐embedded and stained preparations of insect epithelia,^146^ spermatids,^147^ blood platelets,^148^ and neuronal cells.^149^ However, the existence of the lumenal densities was largely thought to be an artifact of the staining procedures. Moreover, there was no evidence of any electron‐dense material in the lumen of in vitro‐nucleated MTs.^117^

Cryo‐ET of frozen sections of cells confirmed the existence of these particles in the MT lumen. In neuronal cells, discrete, closely spaced globular particles of 6–7 nm in diameter were observed^117^ (Figure 5d). The typical distances between particles were apparent multiples of 4 nm, ranging from 8 to 20 nm (Figure 5d). Since 4 nm corresponds to the size of a tubulin monomer, it suggested that lumenal particles decorate the tubulins along the MT wall. Subtomogram averaging revealed points of contact between lumenal particles and the MT wall.^117^ The existence and frequency of lumenal particles was found to vary between cell types, with neuronal cells having the highest abundance.^117^ Depolymerizing MTs contain more lumenal particles as compared to growing MTs, with a minimal interparticle distance of ~8 nm, and have the same “beaded fiber” appearance as observed in conventionally prepared EM specimens.^150^

Identification of intralumenal components is challenging. Recent reports provided evidence for the presence of tubulin acetyltransferase (TAT).^151^ TAT is known to transfer an acetyl group from acetyl‐CoA to the Lys40 of α‐tubulin at the lumenal side^152^ and to regulate MT stiffness,^153^ which is thought to be useful in mechanosensory and cilia functions.

Another occurrence of lumenal material was observed in the singlet region of human spermatozoon tails by cryo‐ET.^142^ In these cells, axonemal MTs display at their distal end a singular interior structure made of diagonal densities with 8 nm periodicity^142^ (Figure 5e). Subtomogram averaging revealed that this lumenal structure, named Tail Axoneme Intra‐Lumenal Spirals, consists of an interrupted left‐handed helix that follows the pattern of the MT lattice^142^ (Figure 5e).

A recent cryo‐ET study of chemically induced, MT‐based cellular projections in HAP1 cells exposed the presence of actin filament segments in the MT lumen^143^ (Figure 5f). Although the physiological relevance of this cell‐based extrusion system remains to be established, it exemplifies the power of cryo‐ET to discover unexpected structures inside cells.

### 3.6 *Structure of the MT plus end*

**FIGURE 6:**
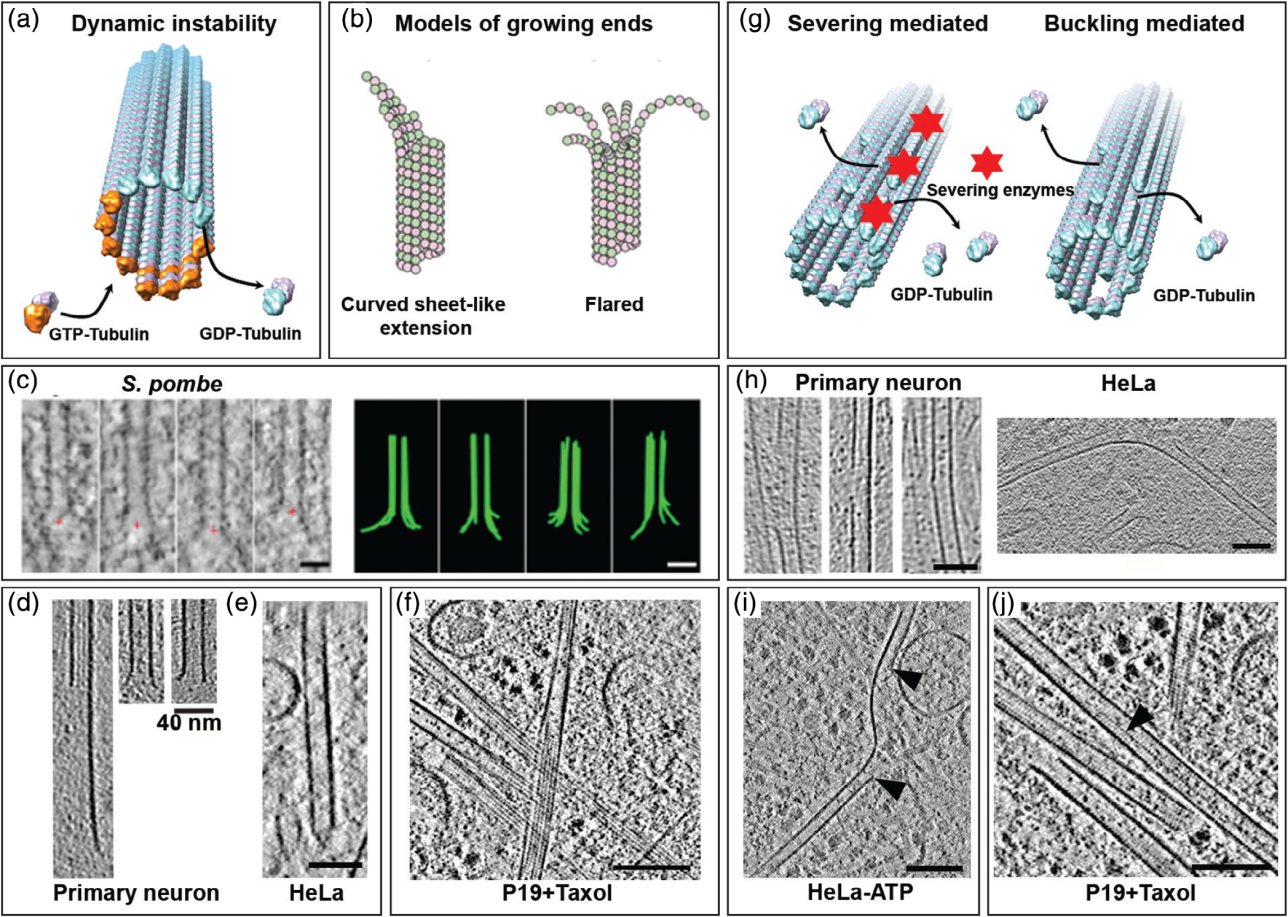
Lattice plasticity of microtubules (MTs) revealed by cryo‐electron tomography. (a) Schematic representation of MT dynamic instability. (b) Proposed models of MT growing ends. Reproduced with permission from reference [155], copyright (2018) Rockefeller University Press. (c) Flared morphology of growing MT plus ends observed in *Schizosaccharomyces pombe* cells. Scale bars: 50 nm. Reproduced with permission from reference [156], copyright (2018) Rockefeller University Press. (d) Curved extension (left), flared (middle), or blunt (right) MT plus ends observed in hippocampal neurons. Reproduced from reference [157]. (e) Capped MT plus end in a mitotic HeLa cell. Scale bars: 40 nm. (f) Sheet‐like extensions of MT plus ends observed in a Taxol‐treated HeLa cell. Scale bars: 100 nm. (g) Proposed mechanisms of lattice defects in MTs. (h) Lattice defects observed in primary neurons (reproduced from reference [157]) and a HeLa cell (reproduced from reference [123]). Scale bar: 40 nm. (i) Protofilament (PF) segments lost in an adenosine triphosphate (ATP)‐depleted HeLa cell indicated by black arrowheads. Scale bar: 100 nm. Reproduced from reference [123]. (j) 13‐to‐14 PF transition (black arrowhead) observed in a Taxol‐treated cell. Scale bars: 100 nm. GTP, guanosine triphosphate; GDP, guanosine diphosphate

MTs exhibit dynamic instability that forms the basis of most MT functions including force generation^154^ (Figure 6a). MTs have the ability to grow or shrink at their plus ends by the net addition or loss of tubulin dimers. Dynamic instability results from the intrinsic GTPase activity of the tubulin dimer inside the polymer.^158^ Conventional EM and later cryo‐EM studies on reconstituted MTs have shown that growing MT ends are structurally heterogeneous^34^ (Figure 6b). Most of them have either a sheet‐like extension that curves away from the long axis of the MT or blunt ends.^159^, ^160^ In contrast, shrinking ends are frayed.^161^ Based on their sheet‐like appearance, the “closing sheet model” for MT polymerization has been proposed where long sheet‐like PFs curl up and form a tube.^159^ Several ET and cryo‐ET studies have challenged this model.^162^, ^163^ It was suggested that the in vitro relationship between MT plus end structure and dynamics is not necessarily identical to the situation in vivo where MT dynamics is regulated by a variety of MAPs.^164^ Elucidating MT end structure in cells can therefore help develop mechanistic models for MT dynamics.

ET studies of MT plus ends in sections of freeze‐substituted cells provided new insights and changed the way we understand MT plus end dynamics. In *S. pombe* cells, when MTs are allowed to repolymerize growing MT plus ends display largely a flared morphology.^165^ McIntosh et al. observed short curved extensions at the ends of growing interzone anaphase MTs in cells from five species^156^ (Figure 6c). They have shown that the extensions are curled PFs growing independently of one another and away from the MT axis. Because the curvatures of PFs were similar between growing and shrinking MTs (being the most curved at the PF tip), they suggested that guanosine triphosphate (GTP)‐bound tubulin is bent in solution and must uncurl to form the MT polymer.

Recent cryo‐ET studies showed curved extensions at the growing MT end in a variety of cells, including plasmodium sporozoites,^166^ fibroblasts,^118^ and hippocampal neurons^157^ (Figure 6d). The observed structures were mostly flared and, sometimes, capped (Figure 6e), sheet‐like, or blunt. The sheet‐like extension was also observed in Taxol‐treated cells (unpublished data; Figure 6f).

Altogether, these studies emphasize the importance of exploring MT structure in its native environment. Cryo‐ET holds promise for the structural determination of cellular MTs including their plus end and end‐binding partners.

### 3.7 *Lattice defects*

Apart from dynamic instability that creates structural heterogeneity at the plus end, in vitro EM studies have revealed the presence of a wide range of irregularities in MT core lattice structures, commonly known as lattice defects^167^ (Figure 6g). These include variations in PF number within a MT^168^ as well as between MTs.^139^ Recent in vitro studies have shown that the MT lattice could also lose a tubulin dimer or several of them, thereby creating a nanoscale hole in the lattice. These defects are hypothesized to arise from cross‐sectional flattening due to mechanical bending,^169^ packing defects,^167^ or the activity of severing proteins^170^ (Figure 6g). In vitro lattice vacancies and dislocations weaken the ability of MTs to withstand compressive forces^171^ and has been shown to affect the efficiency of kinesin‐based transport.^172^ However, in vivo relevance of lattice defects remained obscure.

Recent cryo‐ET studies revealed MT lattice defects of various sizes in neurons^157^ and HeLa cells^123^ (Figure 6h). Because these defects occurred in strongly buckled growing MTs, a causal relationship between MT bending and lattice defects was proposed, although an action of MT severing enzymes could not be ruled out.^123^ In ATP depleted cells, the loss of multiple PF segments was observed (Figure 6i) and may be a long‐lived intermediate resulting from the incomplete action of severing enzymes.^123^ Irrespective of their origin, all these defects could contribute to the apparent strong curvature of MTs in situ. Growing evidence suggests that lattice defects are exploited as lattice control points where fresh GTP‐tubulins can be introduced and thereby rescue/regrow MTs.^173^

PF transition within one MT has only been reported in vitro.^168^ Recent in situ cryo‐ET work showed that it occurs in Taxol‐treated cells, with a transition from 13 to 14 PFs (unpublished data; Figure 6j).

## 4 NANOSCALE ARCHITECTURE OF THE IF CYTOSKELETON

IFs represent a major and structurally diverse group of nonpolar cellular filaments.^174^ They were originally referred to as IFs because their diameter is intermediate between the diameters of actin and myosin filaments forming myofibrils in muscle cells.^18^ IFs are made of smooth PFs composed of antiparallel arranged α‐helical coiled‐coil bundles flanked by small globular domains at either end.^175^ These features make it challenging to study IFs using EM‐based averaging procedures.^17^, ^42^, ^44^

The 3D architecture of IFs within an intact cellular environment remains poorly studied, with only a handful ET and cryo‐ET studies. Cytoplasmic bundles of IFs were observed in ultrathin sections of high‐pressure frozen CHO and 3T3 A31 cells.^176^ Vitrified IFs have a diameter of 12–13 nm on average,^176^ larger than the values reported with conventional EM methods (~7–11 nm).^177^

Cryo‐EM and ET of vitrified sections of human epidermis permitted to visualize bundles of keratin filaments.^178^, ^179^ Keratin filaments have a diameter of 8–10 nm and a center‐to‐center distance between neighboring filaments of about 15 nm.^178^ They present a central core surrounded by an annular ring composed of PFs in a quasi‐hexagonal arrangement.^179^ Keratin structure is different from the 4‐PFs structure found for vimentin.^47^

**FIGURE 7:**
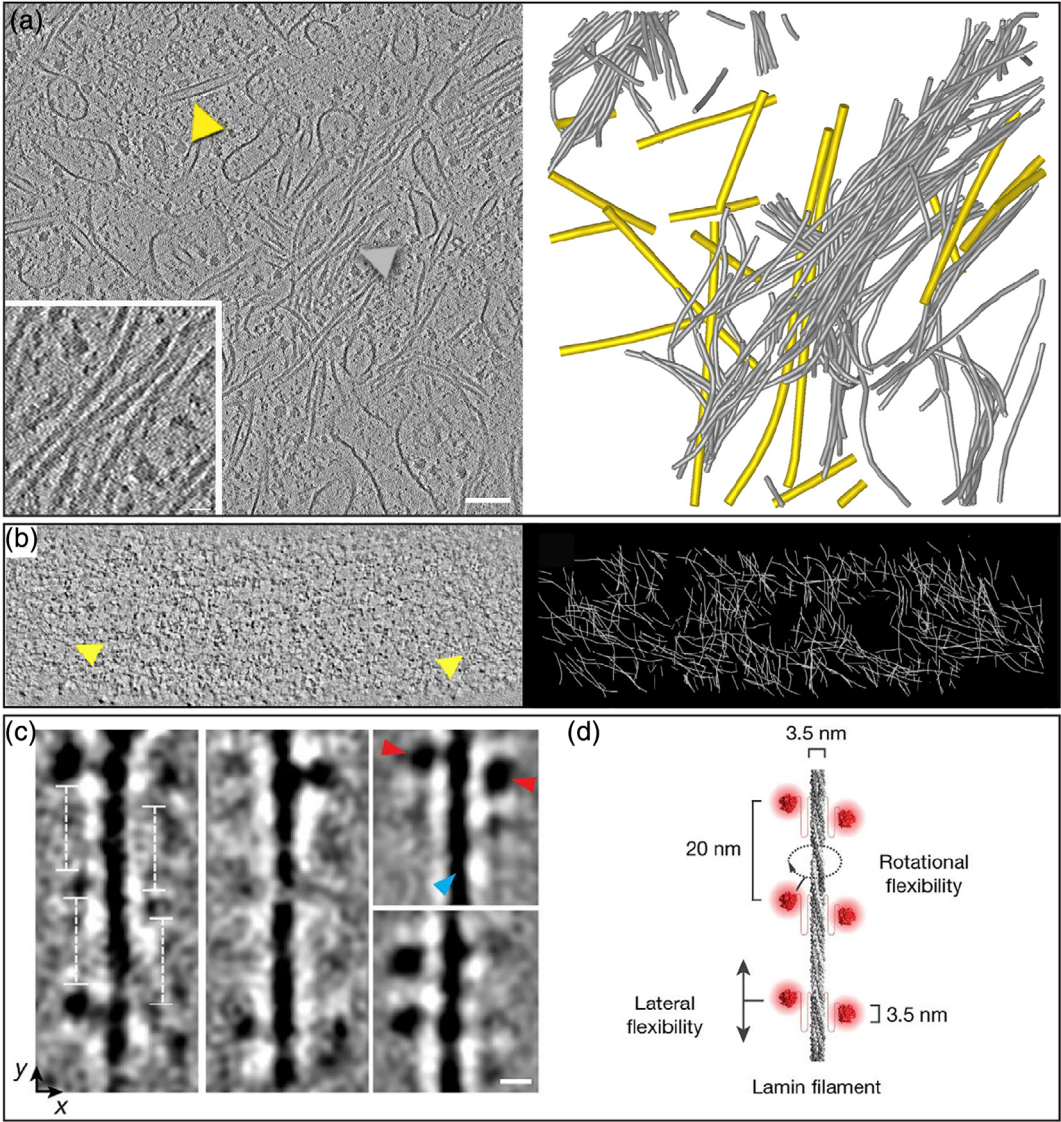
Organization of intermediate filaments (IFs) explored by cryo‐electron tomography. (a) Slice through a tomogram acquired in a focused ion beam‐milled interphase HeLa cell with the Volta phase plate. Inset: Close up on a bundle of smooth cytoplasmic IFs. Arrowheads indicate a microtubule (yellow) and an IF (grey). Cytoskeletal filaments were segmented and displayed using the same color code on the right panel. (b) Slice through the lamina of an interphase HeLa cell. Yellow arrowheads indicate lamin filaments. Three‐dimensional network of lamins are represented in white on the right panel. Reproduced with permission from reference [10], copyright (2016) AAAS. (c) Classes of lamin filaments obtained in vimentin‐null MEFs showing repetitive globular domains spaced 20 nm apart along the filament. Scale bar: 5 nm. Reproduced with permission from reference [180], copyright (2017) Springer Nature. (d) Structural model of lamin filaments. Reproduced with permission from reference [180], copyright (2017) Springer Nature

Cryo‐ET exploration of FIB‐milled HeLa cells showed a dense bundled network of IFs at the perinuclear region^123^ (Figure 7a). Diameter and cross‐section analysis suggested that they are vimentin filaments, although proper identification of the IF type requires correlative approaches. Physical connections between IFs and MTs were observed^123^, ^181^ (Figure 4f) and are found to be more abundant in cells depleted of actin filaments.^123^ These contacts are hypothesized to be responsible for transducing compressive forces onto the MT network, inducing MT bending inside cells.^123^, ^182^

Another type of IFs, known as lamins, is a fundamental constituent of metazoan nuclear lamina. In situ cryo‐ET of HeLa cells revealed that these 4‐nm thick filaments are organized in a mesh‐like structure just underneath the nuclear membrane^10^ (Figure 7b). Cryo‐ET of vimentin‐null mouse embryonic fibroblasts showed that individual lamin filaments have a globular‐decorated fiber appearance (Figure 7c,d), which differs from cytoplasmic IFs.^180^

Future in situ cryo‐ET work could help reveal the native structure and organization of the different classes of IFs across cell types and tissues.

## 5 OUTLOOK

Advancements in cryo‐ET technologies have enabled in situ imaging of cytoskeletal networks with molecular resolution. We can now retrieve both structural and spatial information about cytoskeletal filaments within the cell, linking filament structure to supramolecular assemblies. Future studies may bring insights into “forgotten” cytoskeletal elements such as septins and spectrins. They will also open new avenues to understand the architectural and mechanistic links between cytoskeleton and organelles. Cryo‐ET can be expected to become indispensable for exploring the nanoscale architecture of composite cytoskeletal assemblies in diverse biological systems, and help generate quantitative models for cellular biomechanics. It holds promise for understanding the guiding principles that enable the cytoskeleton to orchestrate physiological processes as diverse as nerve conduction and infection.

## AUTHOR CONTRIBUTIONS

**Saikat Chakraborty:** Conceptualization; writing‐original draft; writing‐review and editing. **Marion Jasnin:** Conceptualization; supervision; writing‐original draft; writing‐review and editing.** Wolfgang Baumeister:** Conceptualization; funding acquisition; supervision; writing‐original draft.
